# The Effect of Group Support Skill Training on the Attitude and Self‐Competency of Caregivers’ Coaches

**DOI:** 10.1002/alz70858_102429

**Published:** 2025-12-26

**Authors:** Mengmeng Xia, Xingyu Zhang, Mei Zhao, Haifeng Zhang, Huali Wang

**Affiliations:** ^1^ Peking University Institute of Mental Health (Sixth Hospital), Beijing, Beijing, China; ^2^ Beijing Jiahua Social Work Center, Beijing, Beijing, China; ^3^ Beijing Haidian Psychological Rehabilitation Hospital, Beijing, Beijing, China; ^4^ NHC Key Laboratory for Mental Disorders, Beijing, Beijing, China; ^5^ National Clinical Research Center for Mental Disorders, Beijing, China

## Abstract

**Background:**

To evaluate the effectiveness of caregiver group support skills training on coaches' attitudes towards dementia care and their sense of competence.

**Method:**

A total of 56 participants in the dementia caregiver group support skills training (referred to as “coaches”; 10 male and 46 female) completed assessments of their dementia care attitudes and sense of competence before and after the training. Paired t‐tests were used to compare the differences in scores for dementia care attitudes and sense of competence before and after the training. Multivariate analysis was conducted to explore the demographic factors associated with the changes in dementia care attitudes and sense of competence.

**Result:**

After the training, coaches showed a significant increase in scores for the “hope” and “personhood validation” factors of the dementia care attitude scale, as well as the total scale score (*p* < 0.05). Additionally, scores for the “professionalism,” “relationship building,” “managing caregiving challenges,” and “maintaining personhood” factors of the dementia care competence scale, as well as the total scale score, significantly increased after the training (*p* < 0.05). However, there was no change in scores for caregiving support competence. Multivariate analysis indicated that only the occupation was associated with changes in the total score and “relationship building” factor of dementia care competence before and after the training; no other sociodemographic characteristics were associated with the changes in dementia care attitudes and other factors of competence scale.

**Conclusion:**

Caregiver group support skills training can improve coaches’ attitudes towards dementia care and enhance their sense of competence in caregiving.

